# Stimuli-Responsive Polymers for Transdermal, Transmucosal and Ocular Drug Delivery

**DOI:** 10.3390/pharmaceutics13122050

**Published:** 2021-12-01

**Authors:** Dmitriy Berillo, Zharylkasyn Zharkinbekov, Yevgeniy Kim, Kamila Raziyeva, Kamila Temirkhanova, Arman Saparov

**Affiliations:** 1 Department of Pharmaceutical and Toxicological Chemistry, Pharmacognosy and Botany School of Pharmacy, Asfendiyarov Kazakh National Medical University, Almaty 050000, Kazakhstan; 2 Department of Medicine, School of Medicine, Nazarbayev University, Nur-Sultan 010000, Kazakhstan; zharylkasyn.zharkinbekov@nu.edu.kz (Z.Z.); Yevgeniy.Kim@alumni.nu.edu.kz (Y.K.); kamila.raziyeva@nu.edu.kz (K.R.); kamila.temirkhanova@nu.edu.kz (K.T.)

**Keywords:** stimuli-responsive polymers, transdermal drug delivery, nasal drug delivery, oromucosal drug delivery, ocular drug delivery, mucoadhesive properties

## Abstract

Despite their conventional and widespread use, oral and intravenous routes of drug administration face several limitations. In particular, orally administered drugs undergo enzymatic degradation in the gastrointestinal tract and first-pass metabolism in the liver, which tend to decrease their bioavailability. Intravenous infusions of medications are invasive, painful and stressful for patients and carry the risk of infections, tissue damage and other adverse reactions. In order to account for these disadvantages, alternative routes of drug delivery, such as transdermal, nasal, oromucosal, ocular and others, have been considered. Moreover, drug formulations have been modified in order to improve their storage stability, solubility, absorption and safety. Recently, stimuli-responsive polymers have been shown to achieve controlled release and enhance the bioavailability of multiple drugs. In this review, we discuss the most up-to-date use of stimuli-responsive materials in order to optimize the delivery of medications that are unstable to pH or undergo primary metabolism via transdermal, nasal, oromucosal and ocular routes. Release kinetics, diffusion parameters and permeation rate of the drug via the mucosa or skin are discussed as well.

## 1. Introduction

For the past several decades, there have been many innovations in the field of “smart” polymers based on polymeric vehicles that show enhanced stability, allowing them to withstand extreme chemical and physical conditions and possess flexible structural parameters, as well as deliver the drug in its native structure and release it in response to specific stimuli such as change in temperature, pH, electroconductivity, etc. [1,2] These stimuli-responsive polymers have shown promising results in the treatment of various conditions such as cancer, cardiovascular diseases, infections and others [3,4,5]. There are a number of papers and book chapters devoted to analysis of papers related to drug delivery systems (DDSs) for oral delivery [6,7,8,9]. Moreover, the applicability of polymeric nanoparticles based on copolymers such as polyethylene glycol (PEG)-polylactic acid, PEG-polyglutamic acid, poly(butylcyanocrylate), PEG-β-cyclodextrin, poloxamers (poly-(ethylene oxide)-poly-(propylene oxide)-poly-(ethylene oxide) (PEO-PPO-PEO)), poly(N-(2-hydroxypropyl)methacrylamide) and poly(2-Oxazoline)s and poly(2-Oxazine)s have been widely investigated for intravenous delivery of anticancer drugs [10,11,12,13]. Another field of applied polymer science and nanomedicine attributed to accelerated interests in inhalable polymer-drug conjugates have attracted the attention of researchers. These polymer-drug conjugates change the pharmacokinetic profile of the loaded drug and, therefore, inhaled administration enables the controlled and prolonged treatment of the lungs. As is the case for intravenous, they should be biocompatible and biodegradable [14,15,16,17].

There is an increasing interest in the development of novel DDSs that can be administered via alternative routes such as transdermal, oromucosal, nasal and ocular. The interest in the utilization of these alternative routes is based on their advantages over traditional administration methods such as oral and intravenous [18,19]. Specifically, the alternative delivery routes are non- or minimally invasive, painless and easy to use for patients. It was also proposed that they could potentially improve the bioavailability of various medications [20,21]. However, the wide use of these administration routes is currently limited, mainly due to low absorption of the medications, which is caused by structural and physiologic barriers associated with the delivery routes. In order to overcome these barriers, a number of strategies have been investigated.

For transdermal drug delivery, passive and active methods have been utilized. Passive approaches act by modifying the composition of a drug formulation to enhance its stability and absorption, and involve the addition of such vehicles as liposomes, nanoparticles, nanoemulsions and others [22,23]. Importantly, these vehicles could be programmed to respond to various stimuli including light, magnetic force, change in temperature, acidity and electroconductivity [22]. Active strategies, on the other hand, use external energy such as heat, electric potential, ultrasound and other forms of energy to drive drug formulations through the skin [22,24]. Another class of active strategies, which is used to improve transdermal drug delivery, is based on microneedles and other more sophisticated devices, for instance, wearable and disposable chemical sensors [22]. These strategies can respond to internal and external stimuli and provide a sustained and controlled release of compounds [22].

Drug delivery via mucosa in the oral and nasal cavities also requires optimization in order to enhance drug absorption. At present, strategies to improve oromucosal drug administration are largely based on patches and films [25,26,27,28,29]. Interestingly, it was demonstrated that programmable oromucosal patches can be rapidly fabricated using 3D printing [27]. Importantly, these patches and films can be designed to be smart, i.e., responsive to specific stimuli [28,30]. Nasal drug delivery systems could be enhanced using cationic polymers, thiolated polymers and in situ gels [31,32,33]. Moreover, “smart” strategies have also been applied to nasal drug delivery systems. Specifically, a number of nasal formulations have been designed to be temperature-, pH- and electroconductivity-responsive [34].

For ocular drug delivery, recent advances include various delivery systems such as microneedles, eye implants, polymeric nanoparticles and in situ hydrogels [35]. Microneedles are able to deliver free or encapsulated drugs in a minimally invasive manner (less tissue trauma, less drug dosage and precise localization of the medication) for the treatment of glaucoma, age-related macular degeneration, uveitis, retinal vascular occlusion and retinitis pigmentosa [36,37]. Intraocular implants can also be advantageous compared to traditional methods of drug administration. They can be introduced via pars plana incision and sutured directly to the sclera for long-term attachment. Implants can release a small-molecule therapeutic over the course of months to years and may also reduce the risk of development of ocular infection or retinal detachment by localizing drug delivery (with a low systemic exposure) to the vitreous humor [35,38]. Moreover, for the improvement of ocular drug delivery, polymeric nanocarriers and polymeric in situ gels have been utilized due to the physicochemical properties of polymers, such as molecular weight, charge, hydrophobicity, biocompatibility, gelation properties and/or mucoadhesiveness, which make them a suitable material for a broad range of ocular applications [19,39,40,41,42]. As with the three other drug delivery systems discussed above, ocular drug transfer can be programmed to release compounds in response to a stimulus. To date, a number of temperature-, pH-, ion- and ultrasound-sensitive ocular drug formulations have been reported [41]. In this review, the most recent developments in the field of stimuli-responsive polymeric DDSs for transdermal, oromucosal, nasal and ocular routes are discussed.

## 2. Transdermal Drug Delivery Systems

Despite all the benefits of today’s most frequently used DDSs, such as peroral, there are still a number of limitations, including poor drug stability in the gastrointestinal tract, low adsorption degree due to interaction with food and, in the case of intravenous administration, strong binding with albumin and other components of blood as well as invasiveness [43]. A transdermal drug delivery system (TDDS) is an alternative route in drug administration, which is currently becoming widely investigated in clinical medicine. The delivery of drugs is accomplished through the skin directly into systemic circulation, which helps TDDS avoid needle-based injections and first-pass metabolism [18]. TDDS also provides a controlled release of drugs, minimizing systemic side effects and enhancing efficacy compared to other delivery routes [44]. Various types of TDDS are currently available, including single-layer drug-in-adhesive, multi-layer drug-in-adhesive, reservoir, matrix and vapor patch [45]. Single- and multi-layer drug-in adhesive patches are the most commonly used due to their simplicity and stability [46]. In the single layer patches, the drug is integrated into the adhesive layer, which makes it accountable for both the release of drugs and attachment to the skin, while in the multi- layer patches, there is an additional layer of a drug separated by a membrane [45,47]. The ease of use and the absence of pain allows TDDS to be used in vulnerable patients, such as children and the elderly. However, the full potential of this delivery system is limited by the skin barrier.

### 2.1. Skin Barrier

**Figure 1:**
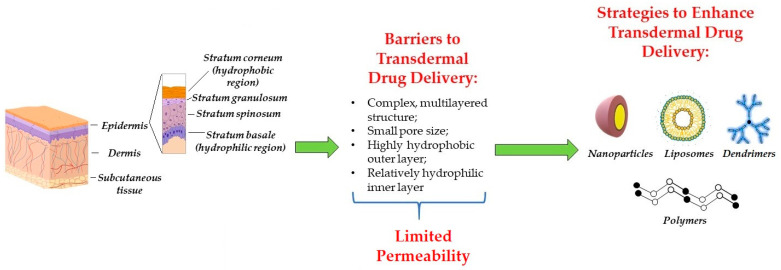
Barriers to transdermal drug delivery and strategies to overcome them. Skin structure and organization represent major hurdles for effective transdermal drug delivery. The multilayered structure of the epidermis and small pore size provide a physical barrier for drug penetration. Furthermore, the highly lipophilic upper layer of the skin prevents the entrance of polar and charged molecules, while the hydrophilic inner layer stops the transfer of hydrophobic compounds. Active and passive techniques have been proposed to overcome the aforementioned barriers. Active strategies use electric, sound, light and mechanical energy to force the penetration of medications through the skin. Passive strategies, in turn, attempt to optimize the composition of drug formulations by adding nanoparticles, liposomes, dendrimers, polymers and other compounds.

Skin is the largest organ of the body, with a surface area of approximately 1.5–2 m^2^. It serves as a physical barrier from external irritants, such as chemical exposure, ultraviolet radiation and pathogenic organisms, and a chemical barrier from internal stimuli, including passage of water and electrolytes [48]. In general, skin consists of three layers: the outer epidermal, middle dermal and inner subcutaneous layers, composed of epithelial and connective tissues [49]. The epidermis is a four-layered stratified structure, composed of the innermost stratum basale (SB), the stratum spinosum (SS), the stratum granulosum (SG) and the uppermost stratum corneum (SC) (Figure 1) [50]. SC, made from 15 to 20 layers of corneocytes filled with filamentous keratin, together with tight junctions in SG, constitutes the key protection layer of the skin [51]. In addition, SC by itself is responsible for the absorption of drugs. Thus, drugs with specific physicochemical properties, such as a molecular weight less than 500 Da, high lipophilicity and a relatively low melting point are allowed to pass through the SC via the passive diffusion [52]. Unlike the epidermis, which is mainly composed of cells, the dermis mainly contains collagen and elastic fibers as well as blood and lymph vessels. The lowermost layer of the skin is made largely from fat, sensory nerves and glycosaminoglycans [53].

The first barrier that a drug encounters when administered is the SC, which allows for the penetration of drugs with low molecular weight and high lipophilicity, such as nitroglycerin, nicotine, scopolamine, clonidine, testosterone, boswellic acid and curcumin [52]. This is because interlamellar regions in the SC contain fluidic intercellular lipids and flexible hydrophobic chains, which are primarily responsible for transepidermal diffusion of the lipids [54,55]. However, the delivery of high molecular weight, hydrophilic or ionic drugs is problematic and may even cause a reversible disruption of the SC layer [52,54]. At the same time, the deeper viable epidermal layer is impenetrable to lipophilic substances. Thus, in order to enter systemic circulation, the drug must pass through hydrophobic and hydrophilic regions of skin, which is not possible for the majority of drugs. For this reason, the main challenge for TDDS is to avoid the barrier effect of SC and to transport the drug into the blood vessels.

### 2.2. Approaches to Overcome the Skin Barrier

Currently, various approaches have been developed to avoid the skin barrier (Figure 1). For example, passive delivery includes the use of vehicles, nanoparticles and nanoemulsion based on chitosan and other polymers [18,56].

Various low molecular weight compounds are also used to increase drug permeation through the skin barrier. Additionally, a transdermal penetration enhancer was used to improve the pharmacokinetics of drug delivery. There are several small organic solvents (DMSO, propylene glycol, laurocapram, 2-pyrrolidone, ethanol, decanol, surfactants, etc.), but we focus on polymer-based systems in this review [57]. The mechanism of permeation using dimethyl sulfoxide (DMSO), which can be explained as a “push–pull effect”, takes place. A rapid permeating enhancer substance (DMSO, limonene, carvone, cineole, α-pinene and 1-dodecyl-2-pyrrolidinone) is added to the donor vehicle. DMSO permeates faster into and through the stratum corneum than Estradiol in the skin; at the same time, DMSO increases the drug’s solubility and a “pull effect” happens, resulting in the diffusion of the drug out of the donor vehicle [58]. DMSO is an organic solvent that has been used for about half a century as a first-choice enhancer for drug permeability [58]. DMSO is a well-known and widely used dermal penetration enhancer with some antimicrobial effects, however, it has disadvantages such as unpleasant smell, local skin irritation and toxic products of degradation. Nevertheless, a number of studies are currently performed by combining DMSO with polymeric DDSs for enhancing drug delivery. The advantages of DMSO are its high polarity and affinity to most drugs, providing excellent solubility of water insoluble substances. For example, the concentration of DMSO increases from 10% to 40%, which results in an increase in bisoprolol fumarate infusion through skin from 5252 to 8335 μg·cm^−2^ in M09-PE and M12-PE PEG-based formulations, respectively [59]. Another study was devoted to Duro-Tak^®^ 387-2510 polymeric sticking agent and stimuli-responsive acrylate copolymer in combination with DMSO, demonstrating a four-time skin permeation increase in Estradiol (Jss = 4.12 μg·cm^−2^·h^−1^) in comparison with the model system containing just DMSO with drug solution (Jss = 1.1 μg·cm^−2^·h^−1^). For example, Estradiol matrix patches containing pH-responsive copolymers of acrylates (13–15 cm^2^, drug capacity 4 mg) Climara^®^ and Menorest ^®^ provided an efficiency release of 50 μg/day for one week. Duro-Tak–DMSO drug load patches with a surface of 1.04 cm^2^ and drug capacity of 0.7 mg were better than similar systems on the market [58]. In vitro permeation studies found that water/oil microemulsion (soybean oil as the oily phase, Brij 58 and Span 80 as surfactants and isopropyl alcohol as a co-surfactant) was better compared to a hydrogel based on polyacrylic acid that was loaded with diclofenac (DC) and DC alone [60]. The dddition of DMSO to the microemulsion enhanced the permeation rate. Thus, the permeability coefficients (Kp) of DC from microemulsion and microemulsion plus DMSO were higher (Kp = 4.9 × 10^−3^ cm·h^−1^ and 5.3 × 10^−3^ cm·h^−1^, respectively) in comparison with the Kp of DS from control (Kp = 2.7 × 10^−3^ cm·h^−1^) and polyacrylic acid hydrogel (Kp = 4.5 × 10^−3^ cm·h^−1^). The results of a paw edema test indicate that microemulsion showed excellent permeation and efficiency, comparable to the microemulsion plus DMSO system [60].

Ali and colleagues investigated a combination of hydrophilic non-ionogenic polymer (PVP 30 kDa) and lipophilic stimuli-responsive polymers (Eudragit RL 100^®^ and Eudragit RS 100^®^) polymers with DMSO (0, 5 and 10% *w*/*w*) for DC delivery [61]. An in vitro pharmacodynamic study illustrated enhanced DC release with an increased fraction of the hydrophilic polymer. TDDS composed of Eudragit RL 100^®^ and PVP in the ratio 40:60 presented the highest drug release (92.45%) with a permeation rate (0.099 mg cm^−2^·h^−1^) and sustained release for 48 h. In vivo monitoring of the DC-loaded Eudragit RL 100^®^ transdermal system revealed a substantially larger degree of inhibition of rat paw edema in comparison with the commercially available formulation of the DC. The authors stated that the formulation is stable and did not show physicochemical interaction for a sufficiently long time (2.52 years) at ambient temperature [61].

In addition to using DMSO as a transdermal penetration enhancer, there are several small organic solvents which are briefly mentioned further in the text, but we focus on polymer-based systems in this review. Elshafeey and colleagues studied the effects of various compositions (cis-oleic acid, Transcutol^®^, PEG 300 NF, (R)-(+)-limonene, (R)-(−)-carvone, cineole, α-pinene and 1-dodecyl-2-pyrrolidinone were Duro-Tak^®^ 87-2074, Scotchpak^®^ polyester) and solvents (DMSO, propylene glycol) for transdermal delivery of fenoterol. PEG 300 was not as efficient for the fenoterol dissolution as DMSO with solubility of 118.5 mg mL^−1^. Fenoterol revealed a longer duration of action than isoprenaline and has less of a side effect on the heart rate. The highest permeability coefficient for fenoterol was observed by transcutol/oleic acid mixture in 1:1 ratio equal to 774.3 cm·h^−1^ × 10^−3^. A comparative analysis of penetration parameters of fenoterol transdermal patches using guinea pig skin treatment with oleic acid and various drug concentrations was performed. The highest rate was illustrated by 12% of fenoterol in cis-oleic acid, with a permeability coefficient of 1188 cm·h^−1^ × 10^−3^. Moreover, it was found that 1-dodecyl-2-pyrrolidnon exhibited excellent performance as a diffusion enhancer of fenoterol [57]. Nevertheless, the use of low molecular weight supplementary substances has some drawbacks and, therefore, there is an opportunity for application of smart polymers. Polymeric nanocarriers are currently of high interest in nanomedicine due to their improved pharmacokinetics, which is expressed in increased membrane permeability and retention effect.

Unique physicochemical properties of nanoparticles, such as size, surface charge, drug-loading efficiency and lamellarity, allow a prolonged and controlled release of an inner substance as well as protect the drug from chemical degradation.

Polymeric nanovehicles can be considered as an alternative, less invasive approach for delivering agents, while avoiding side effects and resistance to drugs and increasing their bioavailability. Co-assembly of amphiphilic poly(ethylene oxide)-block-poly(ε-caprolactone) (PEO-b-PCL) stimuli-responsive polymer with mannosylerythritol lipid (MEL) and YGRKKRRQRRR-cysteamine (TAT)-linked MEL formed a novel polymeric vehicle system that showed elevated cellular uptake through macro- and endocytotic pathways in vitro, and enhanced transdermal delivery in vivo [62]. A recent work used the antimicrobial drug, vancomycin hydrochloride, that was introduced transdermally via the novel systems composed of pH-responsive poly(methylvinyl ether-co-maleic acid) cross-linked by poly(ethylene glycol) dissolving microarray patches (DMAPs) and hydrogel-forming microarray patches (HFMAPs). Ex vivo studies showed that vancomycin hydrochloride was successfully delivered through both HFMAPs and DMAPs with drug penetrating percentages of 46 ± 8% and 8 ± 1%, respectively [63].

Polymeric microneedles are a class of polymer vehicles that is frequently used for transdermal drug delivery. Chen and colleagues used PLA-based microneedles for the delivery of a model dye sulforhodamine B (558 Da) as a fluorescent dye, mimicking drug loading and diffusion parameters. Apart from sulforhodamine B, the coating solution samples also contained polyvinyl alcohol (PVA) for the control of viscosity and sucrose for the stabilization [64]. As much as 22 ng of the compound was delivered with 90% efficiency, while in vivo experiments proved the capability of microneedles for continuous drug delivery and successful skin recovery without any trace of injury. The substance loadings were equal to 12 ng, 14 ng and 18 ng per needle, having heights of 550 μm, 650 μm and 750 μm, respectively [64]. Chitosan is another polymeric material that can be used for a cost-effective, Cross-Over Lines laser engraving technique-based preparation of microneedles in polydimethylsiloxane template and efficient drug delivery through the skin. The nanoneedles have a volume in the range of 20–50 mL and a height of 2–3 mm. Thus, continuous delivery and release of a phenol red dye, mimicking a charged drug, was achieved through the chicken skin by Sadeqi and colleagues [65]. The non-ionogenic polymers PVP and PVA were used for simple, quick and inexpensive fabrication of microneedles by Chen and colleagues, who could ensure an effective skin penetration ability and controllable drug release by the given formulation. Approximately 80% of fluorescein isothiocyanate (FITC), a model drug, was delivered after 48 h through the skin of Kunming strain mice at a ratio of 3:10 PVA to PVP. It is quite unusual that the authors selected this dye, as it is too reactive and will not diffuse via the tissue and mimic drug diffusion. Also, they illustrated an interesting approach of using the FITC@CuS MNs to study the photo-activated transdermal drug permeation, i.e., the skin was irradiated with an 850 nm near infrared laser (1000 mW), reaching 50 °C in 5 min. The results also showed that increased PVA proportion slows down the drug’s release [66]. In addition, it was shown that PVA-based microneedles are capable of enhancing the transdermal delivery of doxorubicin. This illustrated advanced storage conditions under controlled light exposure and that Doxorubicin was significantly more stable in solid PVA microneedles (86 ± 4%) than in an aqueous solution (26.4 ± 1.9%). The drug’s loading to microneedles after the fabrication process was 17.4 ± 1.6%. Gradual release of doxorubicin was achieved by Nguyen and colleagues, showing permeability of 4352 ± 561 ng·cm^−2^ and flux of 226 ± 44 ng·cm^−2^·h^−1^ when applied to the dermatomed human cadaver skin [67]. Apart from that, swelling-modified silk fibroin (SF) microneedles, represented as semi-solid hydrogel with a 50–700 nm pore size, were designed for transdermal drug delivery. A study showed that 2-ethoxyethanol (ECS) modified SF microneedles were able to penetrate into porcine skin in vitro with a depth of ~200 μm and, once inside, formed hydrogels of 50–700 nm [68]. Table 1 summarizes various polymeric microneedles for transdermal drug delivery.

One promising transdermal delivery system is dissolving microneedles (DMN). Once they are applied onto the skin, polymers rapidly penetrate and dissolve, thus releasing therapeutic drugs. Various materials can be used to design such delivery systems, such as polyvinyl alcohol, pH-responsive sodium hyaluronate, chitosan and gelatin [69]. DMN, made of carbohydrate biopolymer pullulan (~200 KDa), showed a good dissolving rate in the skin as well as efficient delivery of low (methylene blue and fluorescein) and high (BSA-FITC) molecular weight substances in a porcine skin model [70]. Rapamycin (RAPA), which can be used to prevent vascular formation during tumor development, was loaded into dissolving polymeric microneedles (RAPA-DMNs) composed of PVP. RAPA loaded into DMNs could penetrate into the skin to a depth of 200 μm. Furthermore, 80% of the drug was released within the first 10 min after the start of treatment [71]. Another group loaded RAPA into phytantriol-based cubosome-like liquid crystalline nanoparticles. RAPA was sustainably released from cubosome-like particles and showed immunomodulatory properties by suppressing natural killer cell proliferation in vitro [72]. Moreover, a 3D printer can be used to design polymer microneedles. Thus, PLA was used as a material for fused deposition modeling 3D printing in a recent study. The characteristics of PLA such as natural degradability and swellability allowed for the construction of a delivery system with needle tip sizes in the range of 1–55 μm [73].

A novel transdermal testosterone system was developed to support the controlled release of hormone for male hypogonadism treatment using cationic forms of poly(vinyl benzyl-N-methyl-D-glucamine) gel with an organic base as a promoter. A “smart” polymeric system of modified poly(vinyl benzyl-N-methyl-D-glucamine) and benzalkonium chloride gel showed acceptable mechanical and rheological properties and enhanced the permeation coefficient (8 ± 2 × 10^−3^ cm·h^−1^ (10% Lim/PG)). The usage of dodecyl sulfate as a co-surfactant for poly(vinyl benzyl-N-methyl-D-glucamine) led to the enhancement of penetration up to 13 ± 5.4 cm·h^−1^, with a total release time of 24 h [74].

Modified molybdenum disulfide with cationic hydroxyethyl cellulose (JR400) revealed reduced toxicity and demonstrated that it can be used as a TDDS for atenolol delivery, the drug prescribed for hypertension. These nanoparticles (NPs) had a flower-like appearance with a diameter of 355 ± 69.3 nm and a drug capacity load of 90.4 ± 0.3%, which provided a sustained release with a 2.3-fold increased penetration of atenolol delivery and did not cause skin irritation [75].

Overall, polymeric nanovehicles are non-toxic, biocompatible and biodegradable delivery systems that have certain advantages over other systems, such as affordability, ease of manufacture and use, ability to load a higher amount of drug and controlled release of active components [76]. Thus, the use of polymeric vehicles for transdermal delivery allows drugs to efficiently penetrate through the skin barrier and to be safely delivered into circulation.

## 3. Transmucosal Drug Delivery Based on Stimuli-Responsive Polymers

### 3.1. Benefits and Limitations Associated with Nasal Drug Delivery

**Figure 2:**
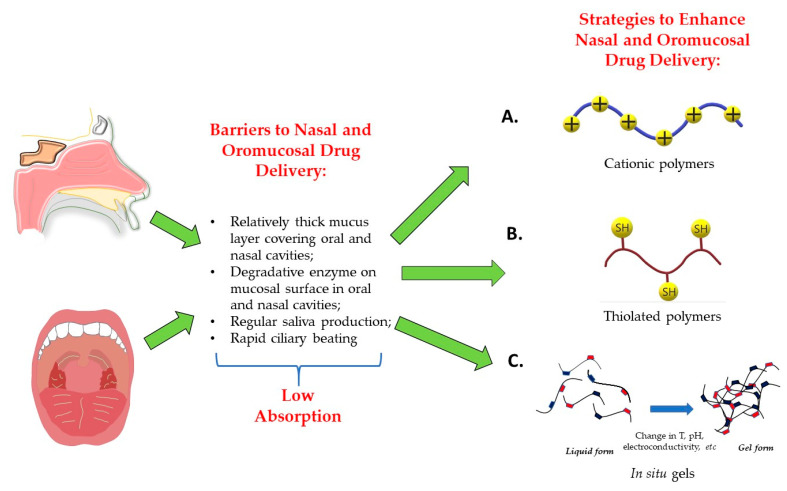
Barriers to nasal and oromucosal drug delivery and strategies to overcome them. Nasal anatomy and physiology significantly limit the absorption of drugs delivered intranasally. The nasal mucosa has a thickness of 5–15 µm and is covered with multiple cilia and degradative enzymes. The ciliary beating and action of enzymes cause rapid clearance of nasally administered medications. Similarly, the oral cavity contains multiple obstacles for drug delivery via an oromucosal route, including a thick multilayered mucosal layer (thickness of 400–700 μm), continuous saliva production and degradative enzymes. In order to enhance the retention and absorption of drugs delivered via the two routes, cationic polymers, thiolated polymers, in situ gels and a variety of nanocarriers have been successfully tested.

Intranasal administration has been shown to be advantageous for the systemic delivery of a number of medications. High systemic concentrations of a compound can be achieved faster with the use of the nasal route compared to conventional methods of drug administration, owing to the fact that the nasal cavity has a large surface area (approximately ~150–160 cm^2^) as well as the presence of ~400 microvilli per cell, a thin epithelium lining, rich blood supply and transmembrane network [77,78,79]. Moreover, drugs administered via the intranasal route do not reach the liver and, hence, escape the first-pass effect, which in turn also contributes to their high bioavailability [33]. Besides enhancing the bioavailability of drugs in systemic circulation, the nasal route offers an opportunity to bypass the blood–brain barrier and improve delivery of medications to the brain [80]. Multiple clinical trials have shown the benefits of a nasal route for the delivery of antidepressants, anticonvulsants and other medications to treat glioblastoma, narcolepsy, opioid overdose and other conditions [81,82,83]. Another advantage of intranasal drug delivery is that nasal mucosa is easily accessible and the administration is painless, relatively straightforward and does not require a trained person to perform the procedure, which favors better patient compliance [32]. Despite the aforementioned advantages, there are several limitations associated with the intranasal route of drug administration, most importantly, low absorption [33]. The absorption of nasally administered drugs is limited due to a mucus layer with a thickness in the range of 5–15 μm, which provides a physical barrier for the diffusion of nasally administered medications [34,84]. In addition, mucus has an overall negative charge, restricting the penetration of anionic drugs. Moreover, regular cilia beating causes mucus to move with a rate of about 5 to 6 mm min^−1^, which results in a rapid particle clearance within 20 min. Another hurdle encountered by nasally delivered drugs is the presence of efflux transporters and degradative enzymes on the nasal epithelium, which actively remove and inactivate absorbed medications [85]. Furthermore, medications which are applied in liquid forms tend to run down the nasal cavity into the pharynx, reducing the amount of the administered drugs. In order to address these challenges, multiple strategies have been proposed, most of which are based on increasing the viscous properties of the solution, which can be accomplished by using novel copolymers (Figure 2).

### 3.2. Approaches to Enhance Nasal Drug Delivery by Using Smart Polymers

Cationic polymers are one group of compounds that have been reported to optimize drug formulations for nasal administration, due to their electrostatic interactions with negatively charged mucins on nasal epithelium [31]. Khutoryanskiy’s research group used the commercial copolymer Eudragit^®^ E PO to mask odors and flavors that were chemically modified in order to improve its mucoadhesive properties and improve its absorption during nasal administration [31]. Specifically, poly((2-dimethylamino)ethyl methacrylate) nanogels (Eudragit^®^ E PO) were modified by the addition of acryloyl chloride to form acrylated polymers. The modified polymers demonstrated greater mucoadhesive properties compared to the original compound, which were assessed by retention studies with fluorescein dye using 10 mg of modified Eudragit^®^ E PO on sheep nasal mucosa. In particular, depending on the degree of acryloylation, two to three times greater quantities of the modified polymers were retained on the nasal tissue after one hour. This study shows that acrylated cationic polymers could enhance the retention of intranasal drug formulations, however, further studies are required to estimate the feasibility of such compounds in vivo. Another strategy to increase retention of nasal formulations on the mucosal surface is to apply pH-sensitive to oxidation thiolated polymers (thiomers) [33]. In contrast to conventional polymers utilized for nasal drug formulations, thiomers are capable of forming covalent bonds with the mucosa through disulfide bridges, and as a result, can enhance retention and absorption. One issue with thiomers, however, is that they tend to form intramolecular disulfide bonds [78]. In order to address this issue, preactivated thiomers, i.e., thiolated polymers, which do not undergo intramolecular reactions, have been developed. Menzel and colleagues designed a novel preactivated thiomer, namely, the co-polymer of 2-((2-amino-2-carboxyethyl)disulfanyl)nicotinic acid (Cys–MNA) and xanthan [78]. The synthesized thiomer possessed superior mucoadhesive properties.

For instance, after two hours, Cys–MNA had a 1.7-fold and 2.5-fold greater mucoadhesion than pH-responsive thiolated xanthan and unmodified xanthan, respectively, when 500 mL of 0.5% (*m*/*v*) solutions were used. Importantly, this is the first study that assessed the nasal safety of a preactivated thiomer using ciliary beat frequency (CBF) of porcine mucosa. Since basal CBF upon the addition of the novel thiomer was 14.0 ± 1.0 Hz, which is comparable to normal CBF, it was suggested that the novel thiomer had not negatively affected ciliary activity and was sufficiently safe. Importantly, the preactivated thiomer synthesized in the study also had properties of in situ gels.

In situ gels represent another class of compounds that can be utilized to enhance nasal drug delivery [32]. These compounds are prepared as liquid formulations, but tend to form gels upon contact with nasal mucosa due to hydrophobic interactions within the gels, which are triggered by various physical (temperature, pH and charge of the mucosa) or chemical factors (for instance, oxidative cross-linking) [86]. Importantly, in situ gels provide an opportunity to achieve sustained and controlled release of a substance [87]. Recently, Jelkmann and colleagues designed a copolymer with improved mucoadhesive properties [32]. In particular, a known carbohydrate-based in situ gel, namely, gellan gum, was modified by reductive amination to increase ionic interactions between the gel and mucosal surface. Examination of mucoadhesive characteristics revealed an up to 32-fold increase in viscosity and a 14-fold greater extended mucosal adhesion time compared to gellan gum, after incubation of the ionic strength and pH-sensitive aminated gellan gum with a concentration of 0.5% (*m*/*v*) for 20 min, suggesting that the synthesized compounds could be useful for improving the retention time of nasal drug formulations. It should be noted, however, that the aminated gellan gum was tested on porcine intestinal mucosa and, therefore, requires further assessment using nasal mucosa [32]. In another study, in situ gels made of pH-responsive carbopol 974P and poloxamer 407 polymers were utilized in order to achieve a prolonged release of buspirone hydrochloride formulated in nanovesicles [87]. The study demonstrated that the nano-vesicular gel formulations could mediate a sustained and controlled release of buspirone hydrochloride after nasal administration. Thus, animal data showed that a novel formulation of the drug had enhanced pharmacokinetic properties, namely, time of maximum concentration observed (T_max_), area under the curve (AUC_0__–∞_) and mean residence time (MRT), in comparison to buspirone hydrochloride preparations without nanovesicles and gels. For instance, the mean AUC_0–24_ for the nano-vesicular gel was 463 ± 10 ng·h·mL^−1^, whereas for conventional oral and nasal formulations of buspirone hydrochloride it was only 67 ± 7 ng·h·mL^−1^ and 142 ± 13 ng·h·mL^−1^, respectively. Moreover, the nano-vesicular gel formulations of buspirone hydrochloride demonstrated a 3.26 times greater bioavailability when administered nasally compared to the regular nasal formulations of the drug. The aforementioned results were evidenced by high-performance liquid chromatography analysis of the quantity of buspirone hydrochloride in blood plasma collected at time points of 0.5, 1, 2, 3, 4, 5, 6, 8 and 24 h after administration and calculating the pharmacokinetic parameters using corresponding software. Nano-vesicular in situ gel preparations can become an efficient platform for controlled and sustained delivery of nasally administered medications.

There are several DDSs illustrating successful applications of modified chitosan. Akhtar and colleagues utilized glycero phosphate with N-trimethyl chitosan, illustrating a sol–gel transition at 32.5 °C and within 7 min as a nasal DDS [88]. The gel forms rheologically synergistic mixtures with mucus and shows a good adsorption level to mucosa. The hydrogel appears to be consistent with its potential use as an in situ thermogelling DDS for intranasal drug delivery. At 35 °C, hydrogel–mucin mixtures illustrate apparent viscosity values more than 350 mPa s, providing better retention on the mucosa. By the change in ratio between glycerophosphate with N-trimethyl chitosan and physiological temperature, one can modulate the gel formation starting from 13 s to a few minutes [88].

Interferon-β (IFN-β) therapy is a treatment used against multiple sclerosis, which has several limitations in terms of a short half-life and reduced central nervous system access. González’s research group developed a novel delivery system based on IFN-β-loaded chitosan/sulfobutylether-β-cyclodextrin nanoparticles to address these issues. Inclusion of IFN-β into the complex with NPs allowed sustained and slow release of the IFN-β. Histological examination of the spinal cords taken from IFN-βNP treated experimental autoimmune encephalitis mice showed that this approach reduced neuroinflammation observed through a decreased number of inflammatory foci, fewer demyelination sites and lower astrocyte and microglia activation. Moreover, it was reported that IFN-β-NPs treatment had a meaningful therapeutic effectiveness, even at reduced concentrations of 78% of the total amount of weekly administered IFN-β [89]. This approach is very promising for delivery of other pharmacologically active peptide-based drugs for the treatment of various brain diseases.

The nasal epithelium hinders delivery of different therapeutic agents as well as antigens used for immunization. Li and colleagues proposed polymer-based intranasal vaccination for human immunodeficiency virus-1 (HIV-1) treatment by an electrostatically bound complex of cationic β-cyclodextrin-polyethylenimine 2k with anionic mRNA encoding HIV gp120 [90]. They observed that the delivery vehicle was able to protect mRNA from degradation in the nasal cavity because, even after 4 h incubation with RNAse, qRT-PCR was still able to detect relatively high gp120 mRNA levels. The presence of β-cyclodextrin with a hydrophobic compartment provided improved mucoadhesive properties of the DDS, allowing longer retention at the nasal compartment. Comparative analysis illustrated that the nasal residence time for naked mRNA and cyclodextrin-polyethylenimine 2k/mRNA accounted for 2 and 3 h, respectively. It was also found that cyclodextrin-polyethylenimine 2k can reversibly open tight junctions, which, in turn, reduced the toxicity of the system and increased production of T and B cells, cytokines such as IFN-γ and interleukin-4, which are responsible for the activation of an anti-HIV immune response [90].

Overall, nasal drug delivery platforms could become an excellent means of systemic administration of various medications as well as improve brain entry of drugs that require crossing the blood–brain barrier, which is especially important for chronic diseases. The efficiency of intranasal delivery can be enhanced by increasing the mucoadhesive characteristics of drug formulations, drug loading level and adaptation of kinetic of release, which, in turn, can be accomplished by introducing novel compounds such as cationic polymers, thiomers and in situ gels. The most recent studies involving these strategies are summarized in Table 2.

### 3.3. Oromucosal Drug Delivery Based on Smart Polymers

Despite the fact that drug administration via oral mucosa is an attractive option, there are several factors that limit drug absorbance at this site (Figure 2). One of the main barriers is the mucus lining itself, which forms an adherent, viscoelastic layer that spreads over the oral cavity. General content of it includes 95–99% of water and 1–5% of mucin, which is a glycoprotein responsible for the barrier properties of mucosa [91]. Due to its adhesive properties, it is able to capture delivered agents and slow down their penetration. However, it can also enhance bioadhesion of the mucoadhesive DDSs and, therefore, can also positively impact a site-specific retention period [92,93]. The thickness of mucosa also plays a crucial role in determining the rate of drug absorption. Buccal and sublingual sites that are commonly used for delivery have a mucosal thickness of 500–800 μm and 100–200 μm, respectively, which makes the first site relatively harder to penetrate. The second issue might be excessive saliva production via the parotid (40%), submandibular (40%) and sublingual glands (10%), which may result in non-uniform distribution of the drug and inhibition of some parts from receiving therapeutic levels of drugs, dislodging of the formulation from applied sites (buccal and sublingual) and premature swallowing of the dosage that consequently reduces bioavailability of drug and requires frequent dosing [92,94,95]. In addition, saliva contains digestive enzymes such as α-amylase, lingual lipase and kallikrein, which can contribute to degradation of the DDSs. Structural specificities of underlying tissues of the oral cavity also explain the barrier function of the oral epithelium against penetration of polar and nonpolar substances. The superficial part of this layer that contains intercellular spaces has materials derived from membrane-coating granules that mainly contribute retarded absorption patterns [96]. Moreover, connective tissue also provides some resistance to lipophilic substances due to high levels of hydration [97].

Compared to other pathways, oral DDSs are much more complex because of physiological, physiochemical, biopharmaceutical and clinical barriers. So, stability, sensitivity, slower clearance and specific site distribution should be provided, because the gastrointestinal tract has solubility at various pH 1.5–7.5, mucus barrier, molecular weight, requirement for fed and fasted state depending on the drug absorptivity and presence of various GI tract enzymes at different sites [98,99]. Orally administered drugs can be absorbed via four types of pathways: transcellular, paracellular, carrier-mediated transcellular and facilitated transport [99]. The oromucosal route of administration is very attractive for delivery of some sensitive drugs as well as proteins, such as IFN, which became the primary focus of many research papers due to the COVID-19 pandemic and other viral respiratory diseases, as discussed earlier. Many antiviral medicinal preparations have sublingual administration [100]. That concludes that the drug has to pass via the 0.1–0.7 mm thick mucus layer. Permeation spots can be divided into sublingual and buccal areas, where the former is easier to permeate than the latter. The drawback of current DDS is related to the generation of saliva by sublingual mucosa leading to a moderately low retention time. Sublingual mucosa is preferable for fast and short duration usages, whereas the buccal mucosa is more suitable for prolonged dosage and onset times. On account of this contrast, the oral cavity is applicable for both local and systemic administration. Dosage forms for the oral cavity are divided into the following classes: gels, ointments, patches and tablets. The most common drug loss may happen by reason of swallowing saliva [101].

Camila Cánepa and colleagues studied IFN-α-2b-loaded pH-sensitive complex of chitosan nanoparticles (IFN-CT NPs), produced by ionotropic gelation of chitosan particles with a size of 36 ± 8 nm and zeta potential of +30 mV, which releases IFN at physiological pH due to weakening of the bonds between chitosan and IFN [102]. The application of this DDS for antiviral activity of IFN-CT NPs in vitro desorption was similar to commercial IFN-α. IFN-CT NPs (0.3 MIU) release in vivo showed detectable levels of IFN-α in plasma after 1 h, while no IFN-α was confirmed using a commercial product [102]. IFN-α is used to treat cancer and viral infections and administered parenterally, as it is unstable in the GI tract and has severe side effects. Imperiale and colleagues produced IFN-α encapsulated with chitosan nanoparticles using an ionotropic gelation method. Results of the experiment showed a good compatibility of nanoparticles with Caco-2 cells, and (PEG)-modified (PEGylated) nanoparticles crossed the intestinal epithelium via a paracellular route. It was found that 19% of PEG CT-NPs and 21% of CT-NPs crossed the Caco-2 monolayer within 4 h, and these formulations have a similar apparent permeability coefficient of 5.531 × 10^−6^ and 6.064 × 10^−6^ cm s^−1^ for PEGylated and unmodified NPs, with no statistically significant differences. This research suggests that nanocarriers have a moderate permeability. Orally administered IFN-α chitosan nanoparticle bioavailability was 56.9 pg·h mL^−1^ in Balb/C mice, reaching a concentration in the plasma similar as after the subcutaneous administration of free IFN-α. It was observed that after the administration of a single dose of 0.3 MIU (0.0014 μg) of IFN-CT-NPs, the concentration of plasma IFN-α reached a maximum concentration of 48 ± 22 pg mL^−1^ [103].

Jøraholmen and colleagues investigated the delivery of IFN-α-2b in PEGylated liposomes with the goal of creating localized therapy against Human Papilloma Virus [104]. In the experiment, INF was used due to its antiviral effect against HPV infected cells. The PEGylation step of the liposome allowed an extension of half-life of the nanoparticle and shifted distribution towards infected tissues due to an increased permeability of capillaries. It also minimized adhesive interactions between vesicles and mucus membrane, which was desired to avoid trapping of vesicles in the mucin fibers. The ability of PEG-coated liposomes to stick onto the mucus layer was estimated on commercially available pig mucin under different pH (4.6, and 7.4), indicating reduced binding affinity of PEGylated liposomes compared to non-coated liposomes and chitosan coated ones. The encapsulation efficiency of INF accounted for 81%. Additionally, the measurement of stability of the PEGylated liposome for leakage of the delivery system once exposed to the testing environment showed only 5.1% of IFN-α-2b detected after 2 h, demonstrating the stability of DDS [104].

The use of unmodified chitosan for IFN delivery has limitations due to its solubility in an acidic medium and relatively poor mucoadhesion properties, therefore, various types of thiolated chitosan can be successfully utilized. Also, the possibility was shown of use of chitosan cross-linked with tripolyphosphate, known as ionotropic gel formation [105]. Treatment of multiple sclerosis requires continuous prolonged administration. Kondiah and colleagues used polyelectrolyte complex N-trimethyl chitosan, PEG-dimethacrylate and methacrylic acid (MAA) for oral delivery of IFN-β. The polyelectrolyte microparticles with 0.5 g/100 mL N-trimethyl chitosan illustrated an INF-β loading efficiency of 53.25% [106]. Fabrice Rose and colleagues designed lipid-polymer hybrid nanoparticles coated with the mucoadhesive polymer glycol chitosan for improved mucosal immune responses [107].

Another study illustrated the approaches of cationic drug metformin delivery via a mucosal route of administration and used various combinations of chitosan-based DDS for diabetes treatment. Retention of the substance in spray dried particles was rapid during the first 5 min and then reached equilibrium within 20 min. Without chitosan spray dried metformin hydrochloride (25 mg), the amount of metformin remaining in particles containing ChitoPharm S (CPS) (3:1 CPS (5 mg/mL) and 1:1 CPS (15 mg/mL)) was larger within a period of time of 1–20 min, although particles containing the lowest amount of CPS (1:3 CPS) illustrated differences only within 5 min. The authors stated that the bioadhesive parameters of spray dried metformin microparticles on porcine buccal mucosa exhibited improved properties after chitosan addition [108].

Klemetsrud and colleagues performed a screening of various polymers, such as chitosan, low-methoxylated pectin (LM-pectin), high-methoxylated pectin (HM-pectin), amidated pectin (AM-pectin), Eudragit, poly(N-isopropylacrylamide-co-methacrylic acid) (p(NIPAAM-co-MAA)) and hydrophobically modified hydroxyethyl cellulose and their effects on cell permeability and interaction with mucin [109]. The authors have studied the effect of the formulations on mammalian cell permeability by evaluating the apparent permeability of mannitol 14C-mannitol via the cell consortium. A viability test of the proliferation of cells after incubation with both chitosan solution and chitosan coated liposomes was 10%. Furthermore, the cell viability of the stratified cells was about 40% after exposure to chitosan coated liposomes. A more compact layer is attributed to stronger interactions, therefore, the pectins are only weakly mucoadhesive. From the other side, the uncoated negatively charged chitosan liposomes are moderately mucoadhesive and the zeta potential of the neutral liposomal formulations altered from neutral to negative after the addition of mucin. The DDSs exhibited no significant effect on cell viability and permeability at the studied concentrations. It was found that the positively charged formulations exhibited the strongest electrostatic interaction, but the negatively and neutrally charged formulations were adsorbed due to hydrogen bond formation, revealing moderate or low sticking. Even though the chitosan-coated liposomes altered the cell viability, this DDS changed the cell permeability, making it an attractive candidate for systemic drug delivery [109]. The ability to adsorb to mucin shows that all the liposomal formulations are promising for oromucosal administration. Layer-by-layer self-assembly films deposition technique is a widely used approach for drug immobilization. The substance benzydamine’s inclusion was performed by alternative dip-coating of corona pretreated PLA into positively charged chitosan or casein solutions, and was then cross-linked by glutaraldehyde/sodium or tripolyphosphate or calcium chloride. This DDS of multilayer polyelectrolyte films was designed for buccal delivery of benzydamine [110].

Another smart commercially available polymeric system, Eudragit^®^ RS 100 (CAR-NC), was used for the formation of nanocapsules of poly(ε-caprolactone) (CAR-LNC) for carvedilol delivery, and it was used to treat heart failure, hypertension and coronary artery diseases. Nanocarriers have a positive charge for CAR-NC and a negative charge for CAR-LNC, illustrating mucoadhesive properties. Encapsulation effectiveness was about 87% and 99% for CAR-NC and CAR-LNC, respectively. It was shown that carvedilol was able to penetrate through the sublingual mucosa [111]. It is a quite novel direction to use okra biopolymer and moringa gum in combination with hydroxypropyl methylcellulose (HPMC), and pullulan as DDS was designed. The disintegration time was less than 0.5 min and the drug content consistency was 98–102% for film formulations possessing superior mechanical properties [112]. Lercanidipine (LR) (611 Da), an aromatic nitroderivative drug, is used as a vasoselective dihydropyridine calcium antagonist for the treatment of hypertension and angina pectoris that should be delivered in a strictly controlled mode. To increase its pharmacokinetic profile, fast dissolving oral films (FDO) were obtained utilizing an evaporative antisolvent precipitation method [113]. The advantage of this DDS over the previously designed one is that nanosuspensions of lercanidipine with PEG 400 and d-alpha tocopheryl PEG succinate 1000 were utilized as stabilizers for PVA, and hypromellose was utilized as the main component of FDO without lyophilizing or spray drying. Superior disintegration and permeation properties of nanoparticles were confirmed by in vitro dissolution experiment, and 4.5-fold better ex vivo drug diffusion was exhibited from formulation through porcine buccal mucosa. PVA in LR-FDO2 illustrated lower crystallinity of matrix and superior physicochemical properties as well as mechanical properties and in vitro lercanidipine release. The steady state flux of the substance through porcine buccal mucosa equaled 0.71 μg·cm^−2^·min^−1^ for the control plain drug and 3.2 ± 0.4 μg·cm^−2^·min^−1^ for oral film (LR-FDO2). The apparent permeability coefficient and diffusion coefficient for plain drug and LR-FDO2 were estimated as 1.78 × 10^−4^ cm·min^−1^ and 2.78 × 10^−6^ cm^2^·min^−1^, and 8.0 × 10^−4^ cm·min^−1^ and 1.2 × 10^−5^ cm^2^·min^−1^, respectively [113]. Overall, fast dissolving polymer-based DDSs or nanocarriers are a prospective approach not only for the treatment of oral bacterial infections, but also for delivery of protein and peptide-based immune stimulating drugs due to a noninvasive route of administration, convenience for patients and relatively high adsorption efficiency of drugs in native state. Table 3 summarizes various approaches to enhance oromucosal drug delivery.

## 4. Ocular Drug Delivery Systems

### 4.1. Ocular Barriers

The eye can be generally divided into two segments: the anterior (cornea, conjunctiva, iris, ciliary body, lens and aqueous humor) and posterior (sclera, choroid, retina and vitreous body) segments [114,115]. Together, these anatomical structures form ocular barriers that define ocular microenvironment and integrity of ocular cells and tissues, protecting the eye and maintaining its homeostasis. However, these barriers can strongly limit drug permeation, resulting in decreased bioavailability of drugs in the eye [116].

The first barrier that drugs have to pass through is the tear film, a thin fluid layer forming the interface of the ocular surface. It is responsible for environmental and immune protection, production of tears (about 1.2 microliters per minute) and their evaporation and drainage [117]. However, it can also act as a barrier for topical application of drugs. Reflex stimulation caused by drug application increases the lacrimation rate from 1.2 microliters to 300 microliters per minute [114], leading to fast drainage of drugs. It is known that a large portion (50–100% of the dose) of topically applied active pharmaceutical ingredients in the tear are lost to systemic circulation, mainly through naso-lacrimal duct drainage [40,118,119]. Moreover, bioavailability of the drug to cornea is also impacted by its affinity for the lipid environment of the outer layer of the tear film [115,120].

Another barrier for drug delivery might be eye cornea. The cornea is a transparent avascular tissue that covers the outer surface of the eyeballs. It consists of six layers with different polarities for each layer [114,119]. The first epithelial layer of the cornea is composed of 5–7 layers of uniformly close-packed cells with tight junctions that prevent the entry of chemicals, microbes and drugs [121]. It is estimated that the corneal epithelium prevents the permeability of hydrophilic drugs up to 90%, and about 10% of lipophilic drugs such as dexamethasone-loaded chitosan nanoparticles dispersed within co-hydroxyethyl methacrylate (HEMA)-ethylene glycoldimethacrylate (EGDMA) [115]. The stroma is the thickest layer (90% of the thickness of the cornea) and mostly consists of water, charged and highly organized hydrophilic collagen, glycosaminoglycans and keratinocytes. Therefore, it also inhibits the penetration of highly lipophilic molecules (penicillin, Fungizone, bromfenac sodium and dexamethasone sodium), but allows permeation of hydrophilic drugs, such as streptomycin [122,123,124,125]. Overall, based on their molecular weight, lipophilicity and ionic charge, approximately 5% of lipophilic and 0.5% of hydrophilic molecules can penetrate the cornea and reach the anterior chamber after topical application [115].

The vitreous humor (VH) is a fragile transparent gelatinous substance located between the crystalline lens and the retina, which occupies about 80% of the eye’s volume. The viscoelastic properties of the vitreous serve as a mechanical damper for the eye, absorbing external impacts and protecting the lens and retina from deformation and injuries [126,127]. VH can also act as a barrier for drugs based on their net anionic charge. It was demonstrated previously that the diffusion of cationic drugs (peptides sequences (Glu-Glu-Lys)8, (Glu-Lys)16, (Glu-Lys-Lys)8, (Glu-Lys-Lys-Lys-Lys-Lys)4 and (Lys-Lys-Lys)8 with Mw ~4 kDa) in VH is dramatically suppressed, whereas anionic drugs remain mobile and freely diffuse ((Glu-Glu-Glu)8, Mw ~4 kDa) [128,129].

**Figure 3:**
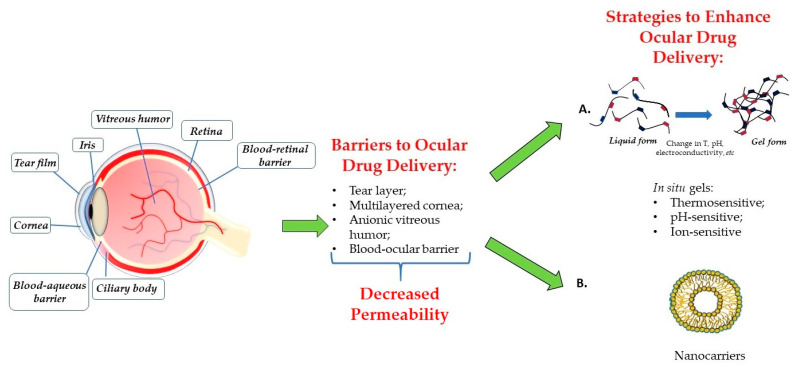
Barriers to ocular drug delivery and strategies to overcome them. The complex structure of the eye reduces the efficiency of ocular drug delivery. Specifically, the tear film, multilayered cornea, anionic vitreous humor and blood–ocular barrier impede the penetration of medications administered via an ocular route. In situ gels, microneedles and nanocarriers have been shown to address the hurdles mentioned above and enhance the efficiency of ocular drug transfer. In situ gels provide an example of “smart” polymers, i.e., they can respond to a variety of stimuli such as change in pH, temperature, electroconductivity, etc.

The blood–ocular barrier is the physical barrier between the ocular blood vessels and the tissues of the eye which prevents the penetration of various substances through it, including drugs. It consists of two main parts: blood–aqueous barrier (BAB) and the blood–retinal barrier (BRB). The BAB is located in the anterior part of the eye between the iris and the nonpigmented ciliary epithelium. At the same time, BRB is located in the posterior part of the eye and is composed of two types of cells: the retinal capillary endothelial cells (inner barrier) and the retinal pigment epithelial (RPE, outer barrier) cells. Both the BAB and BRB possess tight junctions which suppress the penetration of drugs from the blood into anterior and posterior segments of the eye after systemic administration [130,131]. Moreover, the BAB also prevents the penetration of hydrophilic drugs (Pilocarpine hydrochloride 244 Da, Sunitinib malate 532 Da, Sulforhodamine 606 Da, Sulprostone 465 Da) from the blood plasma into the aqueous humor, depending on the molecular weight of the solute. Therefore, a higher molecular weight results in less concentration of solutes penetrated through the BAB into the aqueous humor [132]. In addition, lipophilicity also affects the permeation of drugs through RPE. Lipophilic drugs penetrate the RPE via the transcellular route (the cell membranes of the RPE), whereas hydrophilic drugs mainly pass through tight junctions (a paracellular route). This means that only small lipophilic molecules can permeate the RPE efficiently from blood circulation to the retina [115,132]. Furthermore, the BAB and BRB prevent drug passage to the eye after systemic application by oral or intravenous route. This results in decreased drug bioavailability, and less than 2% of plasma drug concentration reaches the VH. Due to this reduced bioavailability, the administration of high doses of the drug is required to obtain therapeutic concentrations in the intraocular tissues, and it may lead to increased risk of systemic toxicity and severe side effects [133,134]. Overall, the tear fluid layer, eye cornea, VH and blood ocular barriers may interfere with the penetration of various drugs based on their lipophilic, hydrophilic and ionic properties. Thus, DDSs have to pass through these barriers in order to achieve a precise and controlled kinetic of release (Figure 3).

### 4.2. Polymeric Stimuli-Responsive Ocular DDSs

Nowadays, precise and controlled delivery of drugs to anterior and posterior segments of the eye is a major challenge, considering the above mentioned ocular barriers. Ophthalmic in situ gels based on polymers can be utilized to overcome these barriers and has been widely used to develop new polymeric ocular DDSs over the past few years [135]. As discussed above, these compounds are prepared as a liquid solution and tend to transit into gel form due to hydrophobic interactions within the gels, after contact with various physical or chemical factors [86]. Importantly, commonly used ophthalmic in situ polymeric gels provide an opportunity to achieve prolonged and controlled release of the drugs upon contact with physical factors such as temperature, pH and charge of the ocular surface [135,136].

#### 4.2.1. Polymeric Thermosensitive DDSs

Thermo-sensitive hydrogels are in situ gelling systems that undergo phase transition and structural changes in response to temperature, due to an increase in hydrophobicity, formation of intermolecular hydrogen bonds and physical entanglement of polymer chains. These hydrogels are the most investigated stimuli-responsive drugs and are used for the treatment of various ocular diseases, including glaucoma, ocular infections, dry eye syndrome and macular degeneration [137,138]. It is expected that thermo-sensitive hydrogels initiate solution–gel phase transitions in the physiological temperature of the eye (which is around 32–34 °C) and can be stored at a normal room temperature. Currently, there are several popular copolymers, such as poloxamers, natural polymers (cellulose, chitosan derivatives), PLGA, PEG and poly(N-isopropylacrylamide) (pNIPAAM), which are widely used for the preparation of thermo-sensitive hydrogels [137,139].

Recent studies demonstrate that thermosensitive poloxamers (triblock copolymer) can be used as an effective ocular DDS for various drugs by significantly increasing their therapeutic effects compared to marketed treatment. As examples, Poloxamer 407 and poloxamer 188 were used to develop DDS for the delivery of timolol maleate, a potent β-receptor inhibitor, which is widely used as glaucoma therapy for decreasing the production of the aqueous humor. The gel with timolol demonstrated a longer retention time by an increase in T1/2, T_max_ and MRT of TM-TSG (1.85, 1.28, and 1.60-fold, respectively) compared with timolol eye drops with 32 °C gelation temperature. In a rabbit glaucoma model, this DDS resulted in a steady and continuous decrease in intraocular pressure, demonstrating better bioavailability, while timolol eye drops showed a larger fluctuation with a tendency to rebound at the end of the treatment [140]. Another thermosensitive in situ gel, which is based on poloxamer 407 and poloxamer 188, was modified with positively charged carbon dots (C-dots). C-dots were synthesized by the pyrolysis of HA and carboxymethyl chitosan through a one-step hydrothermal method to improve bioavailability of diclofenac sodium (DS), which is used to relieve ocular inflammation. This DDS demonstrated a sustained release of DS for 12 h at 34 °C gelation temperature. Moreover, it increased precorneal retention time 3.45-fold compared to regular DS eye drops, possibly due to electrostatic interaction between positively charged C-dots, nanoparticles and negatively charged corneal epithelial cells [141].

Poloxamers can be further combined with a natural polymer cellulose derivative such as HPMC, which has the ability to increase gel stability and improve drug delivery to the eye by increasing the gel’s viscosity and contact time with the ocular surface, as well as through its interaction with components of the tear fluid [142]. Recently, a thermosensitive gel was prepared by using poloxamer 407 and HPMC polymers by adopting the Box–Behnken experimental design. In situ gel was loaded with nifedipine to decrease the intraocular pressure, which is caused by glaucoma and can lead to severe complications in the eyes. This DDS achieved a 76% drug release after 12 h and was found to possess 30.1 °C gelation temperature and 40 s gelation time. The intraocular pressure was decreased by the gel to 46 ± 3% compared to the marketed conventional eye drops, and required a less frequent application [143]. Moreover, a combination of carboxymethylcellulose, poloxamer 407 and poloxamer 188 was used for preparation of a thermosensitive in situ ocular gel to improve therapeutic efficacy of voriconazole against fungal keratitis. The gel showed a high drug loading capacity (90–97%) with gelation temperature at 29–34 °C. In vivo study demonstrated 8 h of sustained release of the drug from the gel, while voriconazole was not detected in the control group after 4 h, with no sign of ocular damage or clinical abnormality in the cornea, conjunctiva or iris [144].

pNIPAAM is another polymer that demonstrated its efficacy as ocular DDS with a combination of different copolymers including chitosan and hyaluronic acid. A thermosensitive hydrogel based on pNiPAAM/HA was also used for ophthalmic delivery of various drugs. pNiPAAM/HA with ketoconazole (KCL) demonstrated a high loading efficacy (91–96%) due to van der Waals interactions and hydrogen bonding with gelation temperature at 33 °C. In vitro release of KCL using a dialysis membrane method demonstrated that the release profile of a drug through the membrane was 95% in the first 2 h, while in a KCL gel group, only 30% of KCL was released from the gel over the same period of time. Also, in vivo antifungal activity of KCL was higher by almost 30% when compared with the commercially available KCL eye drop in the eyes of animals inoculated with Candida albicans [145]. Moreover, methoxylation effects of benzoic acid derivatives (4-hydroxy-3,5-dimethoxybenzoic acid) were exploited to develop a novel DDS based on chitosan-g-pNIPAAM thermogel with improved antioxidant activities. It was demonstrated that this thermogel loaded with an antioxidant drug (pilocarpine) and inhibitor of histone deacetylases (RGFP966) can prevent development of glaucomatous optic neuropathy by inhibiting oxidative stress and retinal ganglion cell (RGC) degeneration. A single intracameral injection of this DDS, even without pilocarpine and RGFP966, decreased the cup-to-disc (C/D) ratio to 0.78 ± 0.04 compared to 0.93 ± 0.03 after 70 days of the injection, suggesting long-acting antioxidant activities of the modified DDS. Moreover, DDSs loaded with pilocarpine and RGFP966 maintained high RGC density (2532 ± 66 cells mm^−2^), while in a control group, it significantly decreased (347 ± 52 cells mm^−2^) at 70 days after operation, demonstrating great neuroprotective properties of the DDS [146].

Additionally, different combinations of PLGA, aliphatic polyester-based polymer, with other copolymers including poloxamers and PEG in recent studies also showed their potency to increase and sustain the effect of the delivering drugs. PLGA nanoparticles embedded within a glycol-polycaprolactone-PEG and Pluronic F 127 (PEG-PCL-PEG/PLU) thermosensitive hydrogel (nano-thermogel) system were synthesized and employed for the delivery of an anti-angiogenic p11 hexapeptide to the retina. Nanoparticles sized 100–200 nm loaded the peptide with 67% of efficiency. Moreover, this DDS maintained a sustainable release of 70 ± 2% peptide over 60 days at physiological temperature, decreasing the frequency of the injection by two times compared with marketed eye injections (aflibercept (Eylea™)) [147]. Another in situ hydrogel system was developed by using PLGA-PEG-PLGA copolymers to deliver neuroprotective agents for the treatment of retinal degradation. Hydrogel was loaded with anti-inflammatory drugs dexamethasone (0.2%) and ketorolac (0.5%), alone or in combination with the antioxidants idebenone (1 µM) and D-α-Tocopherol PEG 1000 succinate (0.002%). The system demonstrated low polydispersity of 1.22 with gelation temperature at 31–34 °C, and a stable sustained release rate was achieved for 47 and 62 days in dexamethasone and ketorolac groups, with a well toleration rate (85 ± 3.2%) in retinal cells. Moreover, the combination of idebenone and dexamethasone showed great protection against oxidative stress, demonstrating high viability (86 ± 14.7%), while the combination of ketorolac and dexamethasone significantly ameliorated the production of proinflammatory tumor necrosis factor-α [148].

Overall, in situ thermo-sensitive ocular DDSs could become an excellent approach for the systemic application of different lipophilic drugs or substituted with hydrophobic domains that require crossing the ocular barriers. Thus, the efficiency of these thermo-sensitive ocular delivery methods can be increased by using polymers such as poloxamers, cellulose, pNIPAAM, chitosan, PLGA and PEG.

#### 4.2.2. Polymeric pH-Sensitive DDSs

pH-sensitive hydrogels are in situ gelling systems that undergo phase transition and structural changes in response to the changes at the pH level of the environment. The change in pH value can initiate the release of drugs at specific sites in various polymeric delivery systems, mainly via two different methods. In the first method, a large number of ionic side groups (polyacids or polybases) of the main chain of pH-sensitive polymers undergo changes in an ionization state, resulting in solution–gel transition [136,149]. The second method is initiated by the chemical bonds of polymers that are unstable to acid (such as hydrazone, oxime or acetals) or by the use of acid-degradable crosslinking agents to initiate the release of the drugs from the polymeric systems [136,150,151]. In addition, the gradient of pH change can be created using enzymes, for example, urease that hydrolyzes urea to CO_2_ and ammonium shifting the pH. Also, the mild pH change in the environment can be triggered using an easily hydrolyzable agent, such as gluconolacton, shifting the pH to an acidic environment and, therefore, inducing a drug carrier conformation change. As a result, when the pH value of the environment changes, it triggers the cleavage of these chemical bonds, leading to the disruption of an amphiphilic balance of polymers. Such destruction usually leads to the degradation of polymeric nanocarriers, releasing loaded drugs from the system into the surrounding environment [152]. Several pH-responsive polymeric materials were developed as delivery systems in eyes, including polyacrylic acid/carbopol (PAA), cellulose acetate phthalate, polycarbophil and chitosan [136,149,153]. These natural or synthetic pH-responsive polymers can initiate their drug release in a normal ocular surface (pH 7 ± 1.5) and in tear fluid (pH 6.5–7.6) [136,154].

Recently, different combinations of carbopol with other polymers (cellulose and chitosan) were widely used for the preparation of pH-sensitive ocular DDSs. As an example, chitosan nanoparticles were used to load gentamycin for further treatment of bacterial conjunctivitis. Gentamycin loaded chitosan nanoparticles demonstrated entrapment efficiency and loading capacity of 60 ± 1.6% and 34 ± 1.2%, respectively, with a particle size distribution of 135.2 ± 3.24 nm. Furthermore, GTM chitosan nanoparticles were converted into a pH-sensitive sol-gel system using pH-sensitive carbopol 974P, due to polyelectrolyte complex formation. It led to the development of pH induced phase transition in the range of 5 ± 0.36 to 6.5 ± 0.34, which is in the normal scale of ocular tolerance pH (5–7.5), as well as for gelling. It also demonstrated drug content in the range of 97 ± 1.7 to 98 ± 2.06% and exhibited a sustained release (59 ± 1.3%) over 12 h after application. In comparison with marketed eye drops, this sol-gel system showed a significant antimicrobial effect against *Staphylococcus aureus* and *Escherichia coli*, without any morphological changes in histological analysis [155]. Also, an in situ ocular gel was prepared by using Carbopol-974/HPMC polymers loaded with bear bill, an active component of which (Tauroursodeoxycholic acid) showed promising therapeutic outcomes in different ocular conditions such as retinal ganglion, light-induced retinal degeneration, cataract, age-related macular degeneration and retinitis pigmentosa. Despite this, a bear bile extract significantly decreased the gelling ability of the in situ gel, as well as demonstrated a stability at different pH (pH 5.0, 5.5, 6.0, 6.5, 7.0 and 8.0) for up to five days. The gel exhibited a stable sustained release of the drug up to 160 min in vitro and showed increased retention time up to three-fold, compared to the marketed eye drop, in an in vivo experiment on ocular disease-free New Zealand rabbits [156]. Moreover, Allam and colleagues also combined a vancomycin loaded niosome system with Carbo-pol polymer 934P and HPMC for preparation of pH-triggered in situ gelling systems to treat ocular infections [157]. Vancomycin loaded niosomes incorporated into the gel were in a liquid form at the ambient non-physiological conditions (pH 5) and demonstrated longer release (in pH 7.4 of tear liquid) compared to free niosome loaded with vancomycin (39 ± 3.2%, 70 ± 4.7%), while free vancomycin was completely released after 24 h. In an in vivo model of methicillin-resistant *Staphylococcus aureus* (MRSA)-infected rabbits, the antibacterial efficacy of the gel treatment was 180- and 2.5-fold higher compared to the untreated animals and the animals treated with the vancomycin free drug solution, respectively [104]. In addition, betaxolol-loaded niosomes were integrated within a pH-sensitive in situ gel composed of Carbopol^®^ 934P and hydroxyethyl cellulose for an optimal drug delivery. Niosomes, which are loaded into the gelling system, demonstrated a more efficient controlled drug release (89.8%) compared to the drug loaded into the gel (48.6%) or into the niosomes (40.8%) alone in a simulated tear fluid (pH 7.4 and 37 °C) during 24 h. It also demonstrated significant improvements in the bioavailability (280% and 254.7%) and MRT (5.3- and 5.9-folds) of betaxolol compared to the marketed eye drops after ocular application in normal and glaucomatous rabbits, respectively [157].

Furthermore, pH-sensitive polymers can be combined with thermosensitive polymers to prepare dual sensitive hydrogels for the delivery of ocular drugs. Yu and colleagues developed a hybrid nanostructured lipid dual pH- and thermo-sensitive hydrogel (NLC-Gel) for ocular delivery of quercetin (QN), an ocular anti-inflammatory drug. Carboxymethyl chitosan and poloxamer 407 were cross-linked using a naturally occurring cross-linker genipin (GP) to prepare a hybrid hydrogel delivery system. The swelling ratio (SR) of the hydrogel increased as pH and temperature increased, reaching the highest ratio at pH 7.4 and 35 °C, which ameliorates the release of QN in the hydrogel. Moreover, the release of QN from eye drops was 99% within 12 h, while release of QN from the gel was 80.55% within 72 h, demonstrating a better controlled drug release. An in vivo study on rabbits demonstrated that the area under the curve of QN in the gel group was 4.4-fold higher compared to QN in an eye drops group due to a longer precorneal retention time, with no reported toxicity against cells [158]. In another study, pNIPAAM grafted thermo-sensitive heparin and pH-sensitive chondroitin sulfate were loaded with dexamethasone. The system demonstrated a great encapsulation efficiency of dexamethasone phosphate for heparin (60 ± 2.1%) and for chondroitin sulfate (68 ± 1.3%) in the gel. The release of dexamethasone phosphate from the DDS was two-fold slower at 35 °C than at 25 °C (pH 7.4). Moreover, a more prolonged release was detected at a slightly acidic and physiological environment compared to the basic one. Authors indicated that this system can be further used for the ocular delivery of dexamethasone [159]. Thus, pH-sensitive polymers demonstrate promising results as an ocular DDS that provide sustained and controlled release of charged drugs via ion exchange mechanism for the treatment of different ocular conditions. Moreover, their combination with thermo-sensitive copolymers can also increase the efficacy of controlled drug delivery to the ocular site.

#### 4.2.3. DDSs Based on Ionic Strength-Sensitive Polymers

There are a few carbohydrates that have liquid to gel transition in the presence of alkaline monovalent ions (sodium, potassium), therefore, this phenomenon can be successfully utilized for ocular DDSs. Ions present in the eye can be used by DDSs to increase their adhesive properties for providing prolonged and controlled release of the therapeutic agents. Ion-sensitive polymers usually utilize crosslink reactions with ions (Ca^2+^, Na^+^, Mg ^2+^ and K^+^) present in the tear content or ocular surface to enable acquisition of a gel-like structure that successfully covers ocular surface, increasing their time of exposure to the cornea and enhancing the bioavailability of the delivered agent [160,161].

There are several common polymers used for preparation of an ocular in situ gelling “smart” system, including gellan gum, kappa-carrageenan and xanthan gum [136,162]. Recent studies focused on a linear anionic polysaccharide gellan gum demonstrate that it can be used to increase precorneal retention time to enhance the bioavailability of drugs. Bhalerao and colleagues designed an experiment on a system aimed to release levofloxacin using ion-sensitive in situ gelling polymer (gellan gum). In vitro gelling time of the system accounted for less than 15 sec, whereas drug release time was relatively high at 18–28 h. Tested formulations were found to be well-tolerated and a longer precorneal residence time (4 to 8 h) demonstrated prolonged supply of levofloxacin, resulting in increased C_max_ (5564 and 4151 ng/mL), T_max_ (8 and 15 h), AUC0–24 (17,608 and 22,660 h ng mL^−1^) and MRT (8 and 12 h) values for 0.25 and 0.40% gellan gum formulations, respectively [163]. Another study also used a gel-forming solution based on 1,2-distearoyl-sn-glycero-3-phosphoethanolamine-N-[methoxy(PEG)-2000] (PEG-DSPE)/polyoxyethylene esters of 12-hydroxystearic acid (Solutol HS 15) mixed micelles and gellan gum for ophthalmic delivery of curcumin (CUR), a poorly soluble bioactive component. Usage of this mixed micelle and gellan gum combination was justified by the increased stability, solubility and permeability of CUR. For instance, a cellular uptake test showed that PEG-DSPE/Solutol HS 15 mixed micelles were rapidly and in a time-dependent manner taken up by human corneal epithelial cells. Moreover, chemical stability analysis results demonstrated that, in comparison to free CUR, curcumin mixed micelles (Cur-MMs) and mixed micelle in situ gelling system (Cur-MM-ISG) had enhanced CUR chemical stability, and only 1.4% and 1.2% of curcumin degradation was detected within 24 h in these formulations. Irritation examination tests conducted on rabbits showed no effect on the eye, and histological examination detected no changes in the morphological structure of cornea, iris and conjunctiva [164]. In addition, another study conducted by Janga and colleagues proposed ion-sensitive DDSs that form in situ hydrogels of natamycin bilosomes for effective pharmacotherapy. Regarding physicochemical characteristics of this system, it was found that a loading capacity (ratio of entrapped drug and total lipid weight) of the natamycin in bilosomes (NB) was 8.8%, and natamycin content in all the NB formulations (pH 6.2–7.1) was between 90 ± 7.2% and 97 ± 4.1%. A cytotoxicity test also showed that the system was tolerated by corneal epithelial cells and that no changes were observed in histological examination of corneal architecture. The in vitro corneal transport studies supported data on increased permeability characteristics of NT in comparison to control suspension. Moreover, higher mean dose normalized drug levels in the cornea from NB in situ gel with gel residence time of 6 h demonstrated improvements in transcellular penetration of ion-sensitive NB’s [165].

Κ-carrageenan is a natural linear polymeric polysaccharide, and DDSs based on this polymer can undergo sol-gel transition in the presence of potassium ions [166]. Pingfei Li and colleagues used these ions activated in in situ gelling properties of κ-carrageenan to prepare the DDS for delivery of a drug acyclovir via inclusion complex, whose penetration was enhanced by hydroxypropyl-β-cyclodextrin (HP-β-CD) and viscosity agent HPMC [167]. Results of this study demonstrated that κ-carrageenan was more sensitive to potassium and calcium ions and that the viscosity of the gel was able to change upon the addition of cation solution of potassium and calcium of higher concentration (more than 0.05%). Regarding the availability of the drug, due to the delayed release that was exhibited based on κ-carrageenan and HPMC, acyclovir release accounted for 17% within the first half an hour and 80% after 6 h. An irritation test confirmed that the DDS is safe and causes no damage to the eye. Finally, the presence of penetration enhancer HP-β-CD allowed a significant increase in acyclovir absorption, in comparison to conventional eye drops [167]. Moreover, Fernández-Ferreiro and colleagues conducted a similar experiment where they determined surface residence of hydrogel based on κ-carrageenan combined with gellan gum through in vivo testing [168]. After 1.5 h of contact, 77% of the hydrogel remained in the ocular surface, presenting a residence half-life of 262 min, and thus providing evidence of increased bioavailability of the therapeutic agent. Additional studies on ophthalmic safety showed no impact on the tissue, thus no trigger of abnormal blinking that could affect hydrogel removal from the surface [168]. Overall, ion sensitive polymeric DDSs based on gellan gum, κ-carrageenan, xylan gum, pectin and bilosomes can be an efficient approach for the delivery of ocular drugs due to their ability to increase precorneal retention time and bioavailability of the drugs themselves. Table 4 summarizes aforementioned strategies for stimuli-responsive polymers in ocular drug delivery.

## 5. Conclusions and Future Perspectives

Analysis of research from the past decade illustrates a growing interest in designing complex polymeric systems that can provide long-term storage of native drugs, programmed delivery kinetic (laminar gradual release or cyclic) and facilitation of diffusion of the pharmacologically active substances via a cell’s membrane or layer of mucosa. We have only analyzed the routes of drug delivery that lead to a direct local delivery, minimizing the possibility of the drug being degraded by first-pass metabolism in the liver, which takes place after intravenous injection, as well as binding of the drug with albumins. A number of stimuli-responsive polymers can form a complex with drugs via electrostatic and van der Waals interactions and hydrogen bonding, resulting in decreased interaction with mucosa that provides a better penetration ability than the previously widely used DMSO penetration enhancer. Nevertheless, DMSO is still used in combination with polymeric DDSs. Molecular weight and chemical structure (lipophilic and hydrophilic groups) of the drug strongly affects the polymer conformation and rheology of the DDSs, and kinetic release and diffusion can be triggered by some external stimuli such as temperature, ionic strength and pH. A significant breakthrough was achieved in the field of strategies of manufacturing and application of dissolvable microneedles, that have gradually substituted the necessity of using conventional needles or microneedles from inorganic non-biodegradable materials, which have side effects. The application of the transdermal administration route makes it possible to locally deliver highly toxic but effective drugs that cannot be delivered using other routes of administration. Nevertheless, most of the research did not perform in vivo studies of microneedles, but used tests on the heat-treated skin of animals that do not provide comparable pharmacokinetics to human’s skin. There are a number of DDSs based on modified stimuli-responsive chitosan or Eudragit copolymers that were previously used only for oral administration. A quite attractive and promising approach is to design the delivery of immunomodulating peptides, IFN and vaccines via mucosa that allows preservation of their native conformation and high physiological activity. It is interesting to note that, a decade ago, thermoresponsive polymers for drug delivery were restricted by the use of only pNIPAAM, poly((2-dimethylamino)ethyl methacrylate), hydroxyethylmethacrylate-methylmethacrylate and vinylcaprolactam. Nowadays, the focus of researchers has changed to more biocompatible thermosensitive poloxamers, hydrophobically modified carboxymethylcellulose, chitosan glycol, thiolated chitosan and N-trimethylchitosan and other block copolymers that are very useful for the delivery of ocular drugs due to a rapid phase transition, leading to an effective fixation of loaded DDS on the eyes.

A significant trend of research these days is focused on the modification of natural polymers for the creation of DDSs which have several advantages compared to synthetic non-biodegradable polymers, such as a smaller ecological footprint and dealing with wastes as well as utilization of expired formulations. The development of novel thermoresponsive polymers with cyclodextrin or calixarene has a high drug loading capacity. Additionally, the property of solution transformation to gel formation at physiological temperature has high potential for eye drop formulation. Some zwitterionic block copolymers have a unique ability to form micelles in solution and can be utilized as a drug carrier. Beside starch and PVP, there is still not much progress in finding a polymeric system that forms a strong complex with iodine that can be of interest for transdermal delivery. Moreover, insulin delivery using microneedles with modified copolymer phenylboronic acid can be a convenient approach of noninvasive and controlled delivery. Thus, efficient delivery of drugs via ocular, nasal, oromucosal and transdermal routes of administration is important for the effective treatment of various diseases.

## Figures and Tables

**Table 1:** Approaches to enhance efficiency of transdermal drug delivery by using polymeric microneedles.

Formulation	Outcome	Reference
Polylactic acid-based microneedles loaded with sulforhodamine B	Microneedles provided continuous drug delivery and successful skin recovery without any trace of injury	[64]
Poly-vinyl pyrrolidone and PVA microneedles loaded with fluorescein isothiocyanate	Microneedles ensured an effective skin penetration ability and controllable drug release	[66]
PVA-based microneedles loaded with doxorubicin	Microneedles enhanced transdermal delivery of doxorubicin	[67]
Swelling-modified silk fibroin microneedles loaded with 2-ethoxyethanol	Microneedles were able to penetrate into porcine skin in vitro and form hydrogels	[68]

**Table 2:** Approaches to enhance efficiency of nasal drug delivery.

Polymeric System	Formulation	Outcome	Reference
Pre-activated thiolated polymers and in situ gels	Xanthan gum and 2-((2-amino-2-carboxyethyl)disulfanyl)nicotinic acid conjugate	Improved mucoadhesion and stability of liquid formulation compared to either regular xanthan gum or thiolated xanthan gum; no negative effects on ciliary beating	[78]
Cationic polymers and in situ gels	Aminated gellan gum	Increased viscosity, adhesion time and bioavailability compared to non-modified gellan gum	[32]
Cationic polymers	Acrylated Eudragit^®^ E PO (EPO) loaded with fluorescein	Increased adhesion to and retention on mucosa compared to non-modified polymer	[31]
Cationic polymers	Complexes of cationic cyclodextrin-polyethylenimine 2k conjugate (CP 2k) and anionic mRNA encoding HIV gp120	Prolonged retention on nasal epithelium; enhanced humoral and cellular response compared to free mRNA.	[90]
Nanoparticles	Chitosan/cyclodextrin nanoparticles loaded with IFN-β	Improved symptoms in mouse models of autoimmune encephalomyelitis	[89]

**Table 3:** Approaches to enhance efficiency of oromucosal drug delivery.

Strategy of Immobilization	Formulation	Outcome	Reference
(PEG)-modified nanoparticles	IFN-α (PEG)-modified chitosan nanoparticles	Provided detectable levels of IFN-α in plasma within 60 min	[103]
Polyelectrolyte microparticles	Polyelectrolyte complex of N-trimethyl chitosan copolymer methacrylic acid PEGDMA loaded with INF-β	Increased INF-β plasma concentrations compared to the subcutaneous injection formulation	[106]
Cationic polymers	Spray dried particles of chitosan loaded with metformin	Improved encapsulation efficiency for decreased chitosan/metformin ratio	[108]
Liposomes coated with cationic or anionic polymers	Chitosan, low-methoxylated pectin, high-methoxylated pectin, amidated pectin, Eudragit, (p(NIPAAM-co-MAA)), and other polymers	The positively charged DDS exhibited the strongest mucoadhesive interaction	[109]
Polyelectrolyte complexes	Polyelectrolyte complexes of chitosan and casein loaded with benzydamine	Improved drug absorption and release	[110]
Nanocapsules	Nanocapsules based on poly(e-caprolactone) loaded with Carvedilol (CAR) (CAR-LNC) and Eudragit ÒRS 100 (CAR-NC)	Enhanced drug release from the nanocapsules	[111]

**Table 4:** Approaches to enhance efficiency of ocular drug delivery.

Polymeric System	Formulation	Outcome	Reference
Thermosensitive in situ gel with nonionic triblock copolymers	- Poloxamer 407 and poloxamer 188 loaded with timolol maleate, - Poloxamer 407 and poloxamer 188 modified with C-dots for delivery of diclofenac sodium	Increased pre-corneal retention time, bioavailability, steadily decreased intraocular pressure	[140,141]
Thermosensitive in situ gel with nonionic triblock copolymer and semi-synthetic cellulose polymer derivatives	- Poloxamer 407 and hydroxypropyl methyl cellulose loaded with nifedipine, - Poloxamer 407 and carboxymethylcellulose loaded with voriconazole	Demonstrated sustained release of the drug, decreased intraocular pressure and provided high loading capacity	[143,144]
Thermosensitive in situ gel with pNIPAAM copolymer and natural polymers	- pNIPAAM and hyaluronic acid loaded with ketoconazole, - Chitosan and pNIPAAM modified with benzoic acid derivatives loaded with pilocarpine and RGFP966	Demonstrated high loading capacity, sustained release, improved neuroprotective properties and antioxidant activities of the drug	[145,146]
Thermosensitive in situ gel with PLGA and synthetic copolymers	- PLGA nanoparticles embedded with PEG and Pluronic F 127 loaded with p11 hexapeptide, - PLGA and PEG loaded with dexamethasone, ketorolac and idebenone	Increased antioxidative and anti-inflammatory effects of the drug, showed sustained release of the drug and low polydispersity of the gel	[147,148]
pH-sensitive in situ gel with carbopol and natural polymers	- Carbopol 974P and chitosan nanoparticles loaded with gentamycin, - Carbopol 974 and hydroxypropyl methylcellulose loaded with bear bill, - Carbopol 934P and hydroxyethyl cellulose loaded with vancomycin niosomes	Increased retention time and bioavailability, demonstrated high drug content, sustained release and greater effect of the loaded drug	[155,156,157]
pH-sensitive and thermosensitive in situ gelling polymers	- Carboxymethyl chitosan and poloxamer 407 cross-linked with a naturally occurring cross-linker genipin for delivery of quercetin, - Heparin and chondroitin sulfate loaded with dexamethasone	Increased swelling ratio, demonstrated more controlled and prolonged release of the drug due to dual sensitivity, increased precorneal retention time with great encapsulation	[158,159]
Ion sensitive in situ gelling polymer with gellan gum	- Gellan gum loaded with levofloxacin, - PEG-DSPE/polyoxyethylene esters of 12-hydroxystearic acid (Solutol HS 15) mixed micelle and gellan gum loaded with curcumin, - Gellan gum and natamycin bilosomes loaded with natamycin	Demonstrated fast gelling time, high drug content, enhanced solubility and chemical stability, prolonged precorneal residence and release of the drug, increased corneal permeability and persistence on the ocular surface	[163,164,165]
Ion sensitive in situ gel with a natural linear polymeric polysaccharide	- Kappa-carrageenan modified by hydroxypropyl-β-CD and hydroxypropyl methylcellulose for delivery of acyclovir, - Kappa-carrageenan and gellan gum loaded with radiotracers for scintigraphy	Prolonged release of the agent, increased viscosity and absorption of the drug, improved retention time and bioavailability	[167,168]
